# Remarkable Recovery of the Voice

**Published:** 1871-06

**Authors:** 


### REMARKABLE RECOVERY OF THE VOICE.

A singular and very remarkable recovery of the voice is recorded by W. C. Stanley, D. D. S., of Dublin, Indiana, who under date of May 9th, 1871, says: "Miss B. when quite a girl, went to a camp meeting, where becoming excited by the exercises of the occasion, used her voice to excess, and lapsed into a cataleptic state, in which she remained for a considerable length of time. When finally aroused she found her voice and her power of speech completely gone, and for over twenty years she was unable to make an articulate sound. Necessity compelled her to learn to convey her ideas with her fingers to those who understood the deaf and dumb method. Ordinarily she used a slate, and as she could hear perfectly, it was only necessary for her to write.

While in this condition she was under my care two or three times as a patient, and my acquaintance extends over a period of twelve years. About five years ago, a druggist in a neighboring town suggested to her the feasibility of recovering her voice by excessive stimulation with whisky. She consented to make the trial, and became intoxicated to complete stupor. When the effect passed away, Miss B. found her voice entirely restored, and her power of speech unimpaired. Her joy and the joy of her friends can readily be imagined.

There does not seem to be that force and volume in her voice naturally expected from such a person, yet in clearness and precision of tone it is faultless, while her enunciation is so accurate that it is pleasant to hear her converse."

Dr. W. C. Stanley gives the following method of getting creosote, carbolic acid, etc., into the sack of a tooth when there is an opening through the foramen: Fill the root loosely with cotton, saturated with the substance which it is desired to use, wipe out the cavity of decay, and fill with softened gutta percha, leaving it project somewhat from the cavity. Take a large flat instrument and press slowly upon the gutta percha, which will be pressed into the root, and force the fluid through the foramen. By this method I have found it much easier to control the fluid, and prevent its getting upon the soft parts, when there is an opening through the gum, than by any other I have tried, as the pressure can be stopped the instant the fluid makes its appearance at the opening.

